# Harnessing the Rhizosphere Microbiome for Selenium Biofortification in Plants: Mechanisms, Applications and Future Perspectives

**DOI:** 10.3390/microorganisms13061234

**Published:** 2025-05-28

**Authors:** Ruixin Fu, Mengyuan Zhu, Yanrong Zhang, Junmin Li, Haichao Feng

**Affiliations:** 1 School of Biology and Food, Shangqiu Normal University, Shangqiu 476000, China; ruixinfu2022@163.com (R.F.);; 2 College of Agriculture, Henan University, Kaifeng 475004, China

**Keywords:** rhizosphere microorganisms, selenium biofortification, Se-oxidizing bacteria, colonization, crop health

## Abstract

The rhizosphere microbiome plays a critical role in promoting crop health and productivity. Selenium (Se), a beneficial trace element for plants, not only enhances resistance to both abiotic and biotic stresses but also modulates soil microbial communities. Se biofortification of crops grown in seleniferous soils using selenobacteria represents an eco-friendly and sustainable biotechnological approach. Crops primarily absorb selenium from the soil in its oxidized forms, selenate and selenite, and subsequently convert it into organic Se compounds. However, the role of Se-oxidizing bacteria in soil Se transformation, bioavailability, and plant uptake remains poorly understood. In this review, systematic collection and analysis of research on selenobacteria, including both Se-oxidizing and Se-reducing bacteria, are therefore essential to elucidate their functions in enhancing crop growth and health. These insights can (i) deepen our mechanistic understanding of microbially mediated Se cycling and stress resilience and (ii) offer a novel framework for nanomicrobiome engineering aimed at promoting sustainable food production.

## 1. Introduction

Selenium (Se), a trace element with essential biological functions, plays a pivotal role in human health (e.g., participating in the synthesis of glutathione peroxidase) and significantly enhances crop stress resistance and nutritional quality by modulating plant antioxidant systems and photosynthetic efficiency. Selenium exists in nature in four common oxidation states: Se(−II), Se(0), Se(IV), and Se(VI), while Se(−I) is a rare oxidation state seldom encountered in nature [1,2]. Selenium shares very similar chemical and physical properties with sulfur, including identical oxidation states [3]. Notably, the biotoxicity of these selenium species varies considerably: Se(IV) exhibits the highest toxicity, followed by Se(VI), while elemental Se(0) and organic forms (e.g., selenomethionine) show minimal toxicity [4]. Globally, in soil the selenium concentrations exhibit pronounced spatial heterogeneity, with an average of approximately 0.40 mg/kg. Clays, carboniferous and pyritic shales, phosphatic rocks, mineralized organic matter, and coal deposits are highly enriched in selenium, with observed concentrations of Se ranging from 1 to 700 mg/kg [5].

In China, selenium levels range widely (0.01–16.24 mg/kg) but remain generally below the global average in soil [6]. Of particular note, Enshi City in Hubei has received considerable attention as the location of the world’s only known independent selenium deposit, which contains the highest selenium reserves and bioavailable selenium resources globally, earning it the title “The World Capital of Selenium” [7].

In soils, selenium predominantly exists in inorganic forms such as selenite and selenate, which are readily absorbed and utilized by roots [8]. In contrast, organic Se species (e.g., selenoamino acids) are mainly found in anaerobic environments, originating from the microbial degradation of Se-containing organic matter [9,10]. Due to the high chemical similarity between Se and S, plant Se uptake primarily depends on sulfate transporters. For example, selenate (Se^6+^) is taken up by high-affinity sulfate transporters (e.g., Sultr1;1, Sultr1;2, and Sultr1;3) and subsequently translocated to aerial tissues for metabolic assimilation. In contrast, selenite (Se^4+^) uptake likely occurs through phosphate transporters, with most of the absorbed Se(IV) being converted into organic selenium forms within the root cells [7]. Interestingly, a pronounced antagonistic interaction exists between Se and S uptake: elevated sulfate concentrations significantly inhibit Se(IV) absorption, whereas under sulfur-deficient conditions, Se(IV) may promote sulfate transmembrane transport [11]. Moreover, the sulfur-containing amino acid cysteine plays a vital role in the production of volatile Se species in *Pseudomonas tolaasii* under aerobic conditions [12].

**Figure 1:**
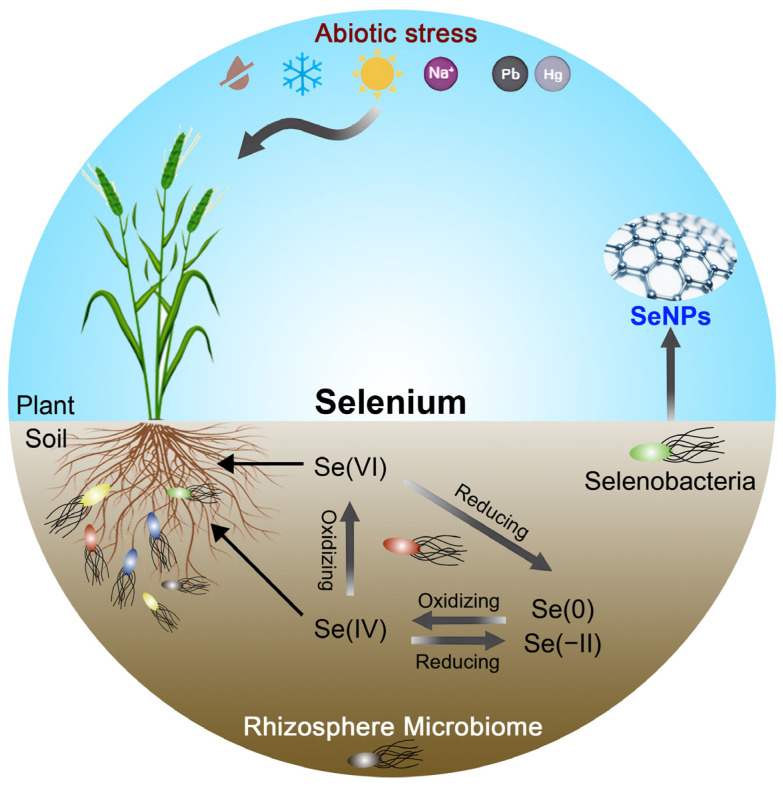
An overview of harnessing the rhizosphere microbiome for selenium biofortification in plants.

Current researches on microbially mediated selenium cycling have primarily focused on reductive processes (Figure 1) [13], while the mechanisms underlying Se oxidation remain poorly understood. Studies have shown that bacterial-mediated selenium oxidation proceeds at remarkably slow rates—three to four orders of magnitude lower than those of reductive reactions [14,15]. In 1972, Torma and Habashi first reported the catalytic oxidation of copper selenide by *Thiobacillus ferrooxidans* [16]. Later, in 1981, Sarathchandra and Watkinson demonstrated that *Bacillus megaterium* could oxidize elemental selenium to selenite, providing a foundational benchmark for subsequent research on selenium oxidation by soil microorganisms [15].

Globally, approximately 15% of the population suffers from selenium deficiency [17], with over 50% of China’s population being affected [18]. Therefore, the World Health Organization (WHO) recommends a daily dietary Se intake of 40 μg for adults [19]. Given the uneven global distribution of soil selenium and the widespread prevalence of selenium deficiency, enhancing the bioavailable selenium content in soils and improving crop selenium uptake efficiency are pivotal strategies for increasing agricultural productivity and food quality. As one of the world’s most important staple crops, wheat is considered an ideal vehicle for selenium biofortification due to its strong selenium accumulation capacity. Selenium biofortification has become a widely adopted agricultural practice and is currently regarded as one of the most effective methods for improving human selenium intake [20]. The rhizosphere microbiome, as the “second genome of plants”, offers a novel route to enhance crop selenium uptake efficiency through the formation of functional plant–microbe holobionts that co-evolve with their host plants. These microorganisms not only represent essential biological resources for ensuring food and nutritional security but also provide foundational support for agricultural technological innovation and the development of modern seed industries.

Soil salinization poses a major ecological threat to global agricultural sustainability. According to the United Nations Educational, Scientific and Cultural Organization (UNESCO), saline-alkali soils cover approximately 932 million hectares worldwide [21]. Reclaiming just 1% of these soils could theoretically boost global grain production by nearly 50 million tons—enough to meet the annual food demand of 120 million people. Recent studies have highlighted that rhizospheric *Bacillus* spp., as key members of halotolerant microbial communities, can alleviate salt stress through multiple mechanisms [22]: (i) secretion of extracellular polysaccharides (EPS) to form protective biofilms; (ii) synthesis of auxins such as indole-3-acetic acid (IAA) to stimulate root development; and (iii) production of osmoprotectants (e.g., proline, trehalose) to maintain cellular osmotic homeostasis. Notably, recent research has shown that selenium nanoparticles (SeNPs) synthesized by Se-transforming bacteria in the maize rhizosphere not only directly enhance plant stress tolerance but also selectively enrich plant growth-promoting rhizobacteria (PGPRs) such as *Bacillus* spp. by modulating root exudate composition [23]. These findings offer new insights into microbe–plant interaction networks and provide a theoretical basis for the targeted manipulation of rhizosphere microecosystems.

Plants exposed to diverse biotic and abiotic stressors actively recruit functionally specialized microorganisms to the rhizosphere to enhance their adaptability. In response to pathogen invasion, plants utilize a “cry for help” strategy to recruit specific beneficial microbes that suppress disease development [24], whereas abiotic stresses (e.g., salinity, drought) selectively enrich the microbiomes capable of mitigating environmental stress [25]. In contrast to these dynamic and context-dependent recruitment patterns, certain probiotic microbes can stably colonize host plants throughout their lifecycle, flexibly modulating their functional traits in response to root exudate composition to meet the host’s physiological needs at different developmental stages [26]. This finely tuned plant–microbe interplay provides important insights for the development of next-generation microbial fertilizers.

In this review, we systematically examine the pivotal roles of microbially mediated selenium (Se) biogeochemical cycling in soil–plant systems, with particular emphasis on how Se-oxidizing/reducing bacteria regulate Se speciation transformations, bioavailability, and crop uptake efficiency. We also focus on the biosynthetic mechanisms of selenium nanoparticles (SeNPs) and their potential agricultural applications, including biofortification and stress mitigation (e.g., salinity, drought). Furthermore, we propose sustainable agricultural strategies based on rhizosphere microbiome engineering, encompassing microbial inoculants and nanofertilizer development.

## 2. Harnessing the Rhizosphere Microbiome to Enhance Plant Selenium Nutrition

### 2.1. Selenium Biogeochemical Cycle

The primary input of selenium into soil appears to be the deposition and mineralization of organic matter [27], though the deposition of selenium from the atmosphere, either as particulate Se(0) and Se(−II) or as Se(IV) and Se(VI) in rainwater, constitutes substantial inputs as well [28]. Soil selenium predominantly exists in five major forms: organic Se, selenides, elemental Se, selenite, and selenate (Figure 1). Among these, selenite (SeO_3_^2−^) and selenate (SeO_4_^2−^) are the principal inorganic selenium species available for plant uptake [8]. Selenite typically accounts for more than 40% of total soil selenium and serves as the dominant inorganic form absorbed by plants. It is primarily found in acidic and anaerobic soils, where it exhibits high solubility and bioavailability [5]. However, selenite readily forms insoluble complexes with iron (Fe) and aluminum (Al) oxides, limiting its effective availability to plants [29,30]. In contrast, selenate is more prevalent in alkaline and well-aerated soils, characterized by its high mobility and plant availability. Nonetheless, due to its extensive water solubility, selenate is prone to leaching losses, resulting in relatively low soil concentrations [30].

Organic selenium, mainly derived from anaerobic microbial degradation, constitutes a crucial bioavailable Se pool. Selenium bound to fulvic acids is readily accessible to plants, whereas humic acid-bound selenium is largely unavailable. Organic selenium, mostly in the Se(−II) oxidation state, originates from the decomposition of plant and animal residues as well as microbial activity [9,10]. Elemental selenium and selenides, typically found in trace amounts in Se-rich mineral deposits, are insoluble in water and thus unavailable to plants [20]. The general order of Se species mobility and bioavailability is as follows: Se(−II) > Se(0) > Se(IV) > Se(VI). Microorganisms are capable of a range of transformations of selenium species, encompassing reduction, oxidation, methylation, and demethylation.

### 2.2. Reduction and Methylation of Selenium

Microorganisms play a central role in driving soil selenium cycling, where the bioavailability of selenium is collectively regulated by three major transformation processes: reduction, methylation, and oxidation (Figure 1). Through the reductive pathway, bacteria convert high-valent selenate or selenite into lower-valent forms such as elemental selenium and selenides. The dissimilatory reduction mechanisms of Se(IV) and Se(VI) exhibit substantial phylogenetic diversity across bacterial species. Even within individual microorganisms, Se(IV) reduction often involves multiple independent enzymatic pathways. Notably, the predominant metabolic end products of both Se(IV) and Se(VI) respiration are SeNPs, which may assemble extracellularly or intracellularly under both aerobic and anaerobic conditions. Several distinct Se(IV) reduction pathways have been studied: (i) the Painter-type reaction involving thiol oxidation, (ii) the thioredoxin–thioredoxin reductase system, (iii) siderophore-associated reduction, (iv) sulfide-coupled reduction, and (v) dissimilatory respiratory reduction. These mechanisms vary in their enzymatic requirements, energy yields, and ecological distribution [31,32].

Generally, Se(VI) and Se(IV) reductases exhibit significant phylogenetic divergence. Intriguingly, all reported respiratory reductases specifically catalyzing the reduction of Se(IV) and Se(VI) exclusively belong to the dimethyl sulfoxide reductase (DMSOR) family and cluster together phylogenetically. These reductases are distinguished by the presence of a molybdenum cofactor (MoCo), a pterin-based prosthetic group essential for their catalytic activity [33]. In most cases, bacterial reduction of selenite to SeNPs occurs in the periplasm and cytoplasm. However, extracellular reduction of Se(IV) has also been observed when bacteria generate and excrete reductive substances [34].

Particularly under high-Se conditions, reductive transformation becomes crucial for microbial detoxification. For instance, certain Se-resistant bacteria specifically reduce selenite into SeNPs to mitigate selenium toxicity [35]. *Bacillus cereus* has been shown to enhance selenium volatilization in soil–plant systems, demonstrating the importance of plant–microbe synergism in selenium phytoremediation [36]. However, the higher the content of selenium in soils where the bacteria were isolated from, the fewer Se(IV)-reducing bacterial species were obtained [13].

Concurrently, the methylation pathway represents another essential route for detoxifying soluble selenate and selenite in soils. Various microorganisms including *Escherichia coli*, the microalga *Chlamydomonas reinhardtii*, phototrophic non-sulfur bacteria, and lactic acid bacteria can convert inorganic Se into volatile methylated species (e.g., dimethyl selenide) [37,38]. These microbial-mediated methylation processes not only supply organic selenium to soils but also represent the dominant route for selenium volatilization and global selenium flux from the soil to the atmosphere [39]. Nonetheless, remediating Se-contaminated soils remains challenging due to the limited understanding of Se biogeochemical dynamics and the high costs of soil remediation.

Studies reveal that under anaerobic conditions, the transcriptional regulator FNR (fumarate and nitrate reductase) in *Enterobacter cloacae* SLD1a-1 specifically modulates selenate reductase activity, facilitating selenium nanoparticle biosynthesis (Table 1) [40]. Remarkably, *Providencia rettgeri* HF16, isolated from coal mine soil, exhibits extraordinary Se tolerance, withstanding selenite concentrations as high as 800 mM—the highest microbial selenite resistance reported to date. This strain not only shows extreme tolerance but also efficiently converts selenite into SeNPs [41]. Mechanistic investigations suggest that bacterial SeNP synthesis involves multiple cellular components, including intra- and extracellular proteins (e.g., flagellin FliC and porin OmpF), lipids, and polysaccharides, which collectively stabilize nanoparticle formation [42]. Furthermore, thioredoxin reductase (TrxR) in *Bacillus* spp. has been confirmed to utilize NADPH as an electron donor for direct Se(IV) reduction into SeNPs [43]. In summary, Se-reducing and methylating bacteria represent valuable biological resources for improving the efficiency of selenium phytoremediation in seleniferous soils.

### 2.3. Oxidation of Selenium

Microbial-mediated oxidation of selenium can significantly enhance the concentration of bioavailable selenium in soils (Figure 1). However, compared with the highly efficient selenium reduction processes, oxidation occurs at much slower rates, and it remains a relatively underexplored area of research [44]. It is intriguing to note that biogenic Se(0) was oxidized more readily than abiogenic Se(0) [15,45]. Recent studies revealed that the *Agrobacterium* sp. T3F4 can oxidize elemental Se into soluble selenite (Table 1). Under soil conditions amended with 5 mg/kg Se(0), strain T3F4 notably enhanced selenium uptake efficiency in pak choi [46]. Subsequently, the same research group isolated four Se-oxidizing bacterial strains from Se-rich soils, each capable of oxidizing selenomethionine, selenocysteine, selenourea, or elemental selenium into selenite. This study offers the first experimental evidence supporting microbial participation in organic selenium oxidation in soils, thereby improving selenium bioavailability and plant uptake. These findings provide critical insights for developing Se biofortification strategies in agriculture [47]. Further investigation is needed to elucidate how microbial activity influences the fate of organic or elemental selenium, which may undergo mineralization or transformation into bioavailable organic forms (e.g., Se-methionine), thus enhancing crop selenium accumulation.

Notably, plants can actively modulate the structure and function of the rhizosphere microbiome through the secretion of root exudates, thereby selectively enriching specific functional microbial groups to increase nutrient bioavailability. Such plant-mediated recruitment often leads to significantly higher bioavailable selenium concentrations in rhizosphere soils compared with bulk soils. Therefore, future research should focus on unraveling the microbial responses to selenium dynamics, including how selenium shapes the microbial community composition and functional gene expression. Meta-omics approaches, such as metagenomics, transcriptomics, and proteomics, will be instrumental in these efforts.

### 2.4. Biosynthesis of Microbial SeNPs

Microbial-synthesized SeNPs are regarded as the optimal selenium form for agricultural and biomedical applications due to their exceptional stability, potent bioactivity, efficient biotransformation, and environmental friendliness. SeNPs exhibit diverse biological activities in organisms, including antioxidant, antimicrobial, antiviral, and antitumor properties [42,48]. Notably, *Bacillus* spp. can reduce selenite to SeNPs via selenate reductase- and glutathione reductase-mediated pathways, employing transmembrane electron transport chains to progressively reduce Se(IV) into elemental Se, which is subsequently secreted as stable extracellular nanoparticles (Figure 1) [49,50]. However, in the presence of Se(IV) and heavy metals such as Pb, Cd, and Cu, microorganisms tend to reduce Se(IV) to nanostructured metal selenides instead of SeNPs [51,52,53].

In soils, SeNPs can function as slow-release selenium sources, providing sustained selenium supplementation for crops and showing promise as sustainable nanofertilizers. Studies have demonstrated that selenium content in wheat grains is strongly influenced by the rhizosphere, where microbial communities modulate selenium speciation and uptake efficiency; SeNP application has been shown to increase selenium accumulation in pak choi by up to 338% [54]. *Chitinophaga* sp. and *Comamonas testosteroni*, both capable of reducing Se(IV), enhanced selenium uptake in rice by solubilizing soil-bound selenium under pot cultivation conditions [55]. Additionally, inoculation of the endophytic Se(VI)-reducing bacterium *Herbaspirillum* sp. into tea seedlings via stem injection significantly increased leaf selenium content compared with uninoculated controls in Se-enriched soils [56]. Under 150 mM NaCl stress, SeNPs synthesized by *B. cereus* were shown to reduce the Na^+^/K^+^ ratio in wheat rhizospheres, significantly improving seed germination rates and mitigating ionic toxicity [57]. Thus, SeNPs play multifaceted roles in enhancing plant stress resistance, including the activation of antioxidant enzymes (e.g., CAT, SOD, GPX) to scavenge reactive oxygen species (ROS) generated under salt stress, thereby protecting cellular metabolism and improving plant resilience.

Beyond stress mitigation, microbial SeNPs can act as “microbial recruiters” that reshape the rhizosphere microbiome. In maize, root-secreted *p*-coumarate activates the *rpoS* gene in *Pseudomonas* sp. ZY71, promoting SeNP biosynthesis. These SeNPs attract PGPRs (e.g., *Bacillus* spp.) in a dose-dependent manner and stimulate biofilm formation, revealing a host-driven strategy for rhizomicrobiome engineering [23]. Similarly, *Bacillus* sp. E5, isolated from wheat roots, converts Se(IV) into organic selenium and SeNPs while alleviating drought stress, thereby enhancing wheat selenium biofortification [58]. The combined application of Se-transforming microbes and SeNPs shows synergistic potential in improving plant health and stress tolerance.

### 2.5. Plant Selenium Uptake Mechanisms

Microbial inoculation has been proven effective in enhancing organic selenium accumulation in crops. The core mechanisms include the following: (i) microbial secretion of organic acids to solubilize elemental selenium, thereby expanding the bioavailable selenium pool; (ii) modulation of plant selenium transporter gene expression (e.g., Sultr and phosphate transporters) by rhizosphere microbiomes to optimize selenium uptake efficiency; and (iii) microbial-mediated transformation of toxic selenite into less toxic organic selenium (e.g., selenomethionine), reducing phytotoxicity. Overall, microorganisms enhance plant growth by improving selenium tolerance and minimizing the accumulation of toxic selenium species (Figure 1).

Plant species exhibit marked differences in selenium uptake and utilization capacities [59]. While low selenium doses promote growth, excessive levels are inhibitory [60]. Currently, agronomic selenium biofortification primarily employs foliar spraying or soil application. Notably, selenium bioavailability is regulated by multiple factors, including soil organic matter content, pH, redox potential, and interactions with other nutrients [30]. Environmental conditions determine selenium speciation: acidic soils favor the formation of insoluble Se(0) and Se(−II), whereas alkaline conditions promote the availability of soluble Se(IV) and Se(VI) [29,30]. Additionally, adsorption by Fe oxides and clay minerals limits Se(IV) mobility, while phosphate (PO_4_^3−^) competes for binding sites, thereby enhancing Se release [5].

Soil selenium application effectively increases both total and bioavailable selenium levels, leading to elevated selenium accumulation in grains, fruits, and vegetables. Comparative studies show notable differences in bioavailability among selenium species: while both SeMet and SeCys2 improve selenium content in winter wheat grains, SeCys2 demonstrates superior enrichment efficiency [61]. In buckwheat, supplementation with 20 μM of Se(VI), Se(IV), or MeSeCys enhances growth, with Se(IV) accumulating more in roots and Se(VI) more efficiently translocated to shoots [62]. Recent research further confirms that selenite application not only optimizes rhizosphere microbial communities and boosts soil enzymatic activity, but also improves salt tolerance in salt-sensitive soybean varieties without yield loss [63]. These findings underscore the importance of precisely tailoring selenium species and dosages for crop-specific biofortification strategies.

**Table 1:** Strain resources of representative selenobacteria.

Selenobacteria	Function	Reference
*Agrobacterium* sp. T3F4	Oxidation of Se(0) into Se(IV); isolated from seleniferous soil.	[46]
Autotrophic bacteria	Oxidation of selenium to selenic acid	[64]
*Bacillus megaterium*	Oxidation of Se(0) to Se(IV); isolated from soil.	[15]
*Dyella* spp. LX-1	Oxidation of SeMet, SeCys2, selenourea, and Se(0) to Se(IV); isolated from seleniferous soil.	[47]
*Thiobacillus ferrooxidans*	Oxidation of copper selenide.	[16]
*Micrococcus selenicus*	Using selenide (Se(−II)) as an electron donor, during aerobic respiration.	[65]
*Rhodanobacter* spp. LX-100	Oxidation of SeMet, SeCys2, selenourea, and Se(0) to Se(IV); isolated from seleniferous soil.	[47]
*Acinetobacter* sp. SW30	Synthesis of gold nanoparticles (AuNPs) and SeNPs.	[66]
*Alcaligenes faecalispine*	Reduction of Se(IV) to Se(0); isolated from the gut of the pine sawyer beetle.	[67]
*Bacillus selenitireducens*	Reduction of Se(IV) to Se(0) or Se(0) to Se(−II); utilizing Se(IV) or Se(0) as an electron acceptor in anaerobic respiration; isolated from Mono Lake, a soda lake in California.	[68]
*Chitinophaga* sp. SE06	Synthesis of SeNPs; the first report of Se reduction by Chitinophaga.	[55]
*Comamonas testosterone* SE26	Synthesis of SeNPs.	[55]
*Enterobacter cloacae* SLD1a-1	Synthesis of SeNPs.	[40]
*Providencia rettgeri* HF16	Reduction of Se(IV) to Se(0); isolated from coal mine soil.	[41]
*Pseudomonas* sp. ZY71	Synthesis of SeNP promoted by *p*-coumarate activates the *rpoS* gene.	[23]
*Streptomyces* sp. ES2-5	Reduction of Se(IV) to Se(0); isolated from a selenium mine soil.	[69]

## 3. Rhizosphere Se-Transforming Microbes in Crop Stress Alleviation

Functional rhizosphere microbes are essential for promoting plant growth and health. The development of these microbes into biofertilizers or inoculants requires effective root colonization as a prerequisite for their beneficial effects [70]. Colonization typically follows a sequential process involving rhizosphere chemotaxis, root surface attachment, and biofilm formation, during which bacteria must evade or suppress plant immune responses [71]. Root exudates play a dual role by serving as both nutrient sources and signaling molecules that mediate microbe–plant interactions [72]. These exudates, mainly composed of amino acids, organic acids, and sugars, act as chemoattractants that guide microbial colonization. Recent studies have shown that the assembly of root-associated microbiomes is determined more by the dominant compounds and their corresponding microbial receptors than by the cumulative effects of multiple exudate components [73,74]. Successful colonizers rapidly utilize root exudates as carbon sources and compete effectively for limited resources such as iron, which is crucial for establishing a stable niche in the rhizosphere [75]. Under environmental stress, plants enhance the recruitment of beneficial microbes through the “cry for help” mechanism [76].

In addition to soluble compounds, root-emitted volatile organic compounds (rVOCs) serve as long-distance signals that modulate microbial behavior. These rVOCs diffuse readily through the soil matrix, promote quorum sensing, attract antifungal microbes during pathogen attack, and facilitate biofilm formation. For instance, the volatile compound dipropyl disulfide released by *Allium* species modifies tomato root exudate profiles, leading to the enrichment of beneficial rhizobacteria [77]. A recent study demonstrated that surfactin production enables *Bacillus subtilis* to stably colonize synthetic microbial communities without altering community composition while significantly reshaping the overall metabolic profile [78]. This finding provides new insights into the design and optimization of microbial agents for sustainable agriculture.

Selenium is a beneficial element for plants that can alleviate adverse conditions such as salinity, drought, high temperature, freezing damage, and heavy-metal stress. Numerous studies have indicated that selenium in the soil can improve the synthesis of plant antioxidant substances, activate antioxidant enzymes, enhance the ability of plant cells to scavenge free radicals, prevent membrane lipid peroxidation, and protect the plant cell membrane system, thereby mitigating the detrimental effects of both abiotic and biotic stresses on plant health [63,79,80,81]. The plant growth-promoting effects of bacteria vary depending on the metabolic capacities and stress tolerance traits of the specific bacterial strains involved [82]. PGPR mediates a wide range of physiological responses in plants, including the biosynthesis of proline and the production of IAA, a key phytohormone that also contributes to bacterial stress adaptation [83]. IAA production notably peaks under drought stress conditions [84]. In parallel, proline functions as a multifunctional osmolyte, maintaining redox homeostasis and acting as a reactive oxygen species (ROS) scavenger [83].

Recently, a novel strategy has been proposed to improve plant selenium uptake while mitigating the absorption of highly toxic metalloids through the application of Se-oxidizing bacteria. For instance, selenium-oxidizing *Agrobacterium* sp. T3F4 significantly reduced arsenic accumulation in *Brassica rapa* L. grown in naturally contaminated soils [9]. The application of selenium can enhance soybean salt tolerance by optimizing the rhizosphere microbial community and improving the salt tolerance of salt-sensitive soybean [63].

## 4. Future Perspectives

In summary, researchers have increasingly focused not only on the remediation of selenium pollution but also on biofortification strategies and dietary supplementation due to the widespread occurrence of selenium deficiency. Establishing a direct biochemical pathway connecting selenium biogeochemical cycling with specific biomarker genes or metagenomic signatures remains challenging. This difficulty arises from the abundance of genes encoding nonspecific selenate/selenite reductases and the currently limited characterization of enzymes involved in both the methylation and oxidation pathways of selenium. In addition, future studies are expected to prioritize elucidating the diverse enzymatic mechanisms underlying microbial oxidation of selenium (e.g., organic Se, elemental Se).

Soil salinization poses a critical threat to global agricultural production, compromising food security, agricultural sustainability, and ecosystem health. Rhizosphere selenium-transforming microorganisms significantly contribute to plant selenium biofortification and salt-alkali tolerance. Specifically, selenium-reducing bacteria synthesize microbial SeNPs, while co-inoculation with selenium-oxidizing bacteria enhances salinity tolerance and selenium content in wheat seedlings. These insights provide a theoretical foundation for (i) utilizing saline-alkali lands, (ii) improving wheat yield/quality via selenium biofortification, and (iii) elucidating rhizosphere microbial symbiosis mechanisms. Such advances are vital for achieving stable microbial colonization and promoting crop resilience.

## Data Availability

No new data were created or analyzed in this study.
